# Effects of supplementing sweet sorghum with grapeseeds on carcass parameters, and meat quality, amino acid, and fatty acid composition of lambs

**DOI:** 10.5713/ab.22.0189

**Published:** 2022-11-14

**Authors:** Jianxin Jiao, Ting Wang, Shanshan Li, Nana Gou, A. Allan Degen, Ruijun Long, Hucheng Wang, Zhanhuan Shang

**Affiliations:** 1 State Key Laboratory of Grassland Agro-Ecosystems, College of Ecology, Lanzhou University, Lanzhou 730000, China; 2 State Key Laboratory of Grassland Agro-Ecosystems, College of Pastoral Agriculture Science and Technology, Lanzhou University, Lanzhou 730020, China; 3 The First Affiliated Hospital of Henan Polytechnic University, The Second People’s Hospital of Jiaozuo, Jiaozuo 454000, China; 4 Desert Animal Adaptations and Husbandry, Wyler Department of Dryland Agriculture, Blaustein Institutes for Desert Research, Ben-Gurion University of the Negev, Beer Sheva 8410500, Israel

**Keywords:** Fatty Acids, Grapeseeds, Meat Color, Small-tailed Han Lamb, Sweet Sorghum

## Abstract

**Objective:**

Sweet sorghum is an important forage crop for ruminants, especially in low rainfall areas. Grapeseeds are an abundant by-product of wine-making and contain bioactive substances that can improve the antioxidant capacity of meat. We examined the effect of sweet sorghum forage with supplementary grapeseeds on carcass and meat quality in lambs.

**Methods:**

Twenty-eight Small-tailed Han lambs (body weight = 19.1±1.20 kg), aged 3 to 4 months, were penned, and fed individually. The lambs were divided into four groups (n = 7 each) and were offered one of four diets: i) sweet sorghum silage; ii) sweet sorghum silage + grapeseeds; iii) sweet sorghum hay; and iv) sweet sorghum hay + grapeseeds. The grapeseeds were added to the concentrate at 6% DM and the diets were fed for 100 d.

**Results:**

Sweet sorghum silage tended (p = 0.068) to increase hot carcass weight, while grapeseeds tended (p = 0.081) to decrease dressing percentage without affecting other carcass parameters. Lambs consuming supplementary grapeseeds increased (p<0.05) meat redness and tended to decrease (p = 0.075) concentration of methionine in meat. Lambs consuming sweet sorghum silage increased (p<0.001) water content of the meat and had a lower (p<0.05) concentration of n-6 polyunsaturated fatty acids (PUFA) and n-6:n-3 PUFA ratio than lambs consuming sweet sorghum hay. Saturated fatty acids content in meat was lowest (p<0.05) in lambs consuming sweet sorghum silage with grapeseeds. Lambs with supplementary grapeseeds tended (p<0.10) to increase eicosapentaenoic acid and docosahexaenoic acid and have a lower thrombogenic index than lambs not consuming grapeseeds.

**Conclusion:**

It was concluded that sweet sorghum with supplementary grapeseeds fed to lambs; i) improved the color of the meat to be more appetizing to the consumer; ii) tended to improve the fatty acids composition of the meat; and iii) lowered thrombogenic index of the meat.

## INTRODUCTION

The development of high-quality roughages and plant-derived additives are important in promoting sustainable livestock production. Sweet sorghum is regarded as a high-quality forage and as a potential alternative to corn. This crop provides a high yield and nutritional value, is drought tolerant [1], and could be crucial in solving the serious increasing forage shortage in China, especially in dry areas [2]. When fed to lambs, sweet sorghum mediated rumen bacteria communities and altered volatile fatty acid concentrations in sheep [3].

Grape pomace, composed of seeds and skins, is a by-product of wine-making and represents about 20% of the grape [4]. It is a potential source of supplementary feed as it contains compounds such as phenolic acids, flavonoids and proanthocyanidins [5], as well as needed fatty acids, amino acids, and minerals [6]. Grapeseeds or grape pomace, as feed supplements, improved lamb production [7], the digestion and utilization of nutrients in steers [8], and the quality of sheep’s milk [9]. However, there is little information available on the effect of grapeseeds on the meat quality of lambs. The present study aimed to fill this important gap. Grape by-products, as a plant-derived additive, could have broad application in the production of organic animal products.

Grape by-products are readily available in China and sweet sorghum is suitable for planting in arid areas of northwest China and is considered a new forage resource. Because of the positive effects of grapeseeds on lamb production, we hypothesized that grapeseeds would improve the carcass and meat quality of lambs. To test this hypothesis, we examined the effects of supplementing sweet sorghum with grapeseeds on carcass parameters, and meat quality, fatty acid and amino acid concentrations of Small-tailed Han lambs.

## MATERIALS AND METHODS

### Animal care

All animal procedures were approved by the Animal Care Committee of Lanzhou University (Protocol approval number SCXK Gan 20140215).

### Experiment design and animal management

The study, a 2×2 completely randomized design, was conducted between mid-August and late-November 2017, at the Xiangtai farm, Dingxi city, Gansu province, China. Twenty-eight male Small-tailed Han lambs (body weight [BW] = 19.1±1.20 kg), aged 3 to 4 months, were penned, and fed individually and assigned randomly to one of four dietary treatments (n = 7 per group). The lambs received alfalfa silage (1% BW on dry matter [DM] basis) and concentrate feed (2% BW on DM basis) plus: i) sweet sorghum silage (SS); ii) sweet sorghum silage + grapeseeds (SSG); iii) sweet sorghum hay (SH); or iv) sweet sorghum hay + grapeseeds (SHG).

All the lambs were offered sweet sorghum silage or hay *ad libitum* and clean water throughout the study. Grapeseeds were dried and ground into a powder, and were added to the concentrate at 6% DM. The sweet sorghum silage, sweet sorghum hay and alfalfa silage were bought from Minxiang Herbage Company (Dingxi, China) and the grapeseeds from Jiabainong Wine Company (Yinchuan, China). The feed ingredients and nutrient composition of the diets are presented in Tables 1 and 2. The sheep were de-wormed (Ivermectin, Zoetis, Suzhou, China) and quarantined for 7 d prior to the study. The lambs were fed two times a day in individual pens at 08:00 and 17:00 for 100 d. Daily feed intake was determined for each lamb over 100 days by weighing the feed offered and then weighing the orts each morning.

### Slaughter procedure and carcass evaluation

At the end of the feeding period, the lambs were fasted for 24 h and deprived of water for 2 h. The lambs were weighed (live body weight, LBW) and then slaughtered in a commercial abattoir according to industry norms. The wool, head, hooves, tail, and viscera (except kidneys) were removed after slaughter and, after 30 minutes, were weighed for hot carcass weight (HCW). Dressing percentage was calculated as: (HCW/ LBW)×100.

The rib eye area was measured at the cross section of the *Longissimus lumborum* muscle of the 12th intercostal space using sulfuric acid drawing paper and was calculated as: (height/2 × width/2)×Π. Backfat thickness on the external surface of the *Longissimus thoracis* (LT) muscle and GR tissue depth at 110 mm from the carcass midline of the dorsal spine between the 12th and 13th ribs were measured using a Vernier caliper. Then the LT muscle between the 12th and 13th ribs was collected to determine meat quality; part of the muscle was stored at −80°C for fatty acid and amino acid profiles.

### Analyses of feedstuffs

The contents of DM, organic matter, crude protein (CP), ether extract (EE), acid detergent fiber ash in feed were measured according to the procedures of the Association of Official Analytical Chemists (AOAC) [10]. Neutral detergent fiber (NDF) was measured with sodium sulfite and amylase added following Van Soest et al [11]. The non-fiber carbohydrate content was calculated by subtracting CP, NDF, and EE from organic matter [12]. The total tannins content in grapeseeds were determined by the Folin-Ciocalteu method as described by Zhang [13].

### Meat quality measurements

Meat color, pH, water content, pressing loss, cooking loss and drip loss were determined of LT tissue. Lightness, redness, and yellowness of tissue were measured using a chroma meter (FRU WR-18; Shenzhen Wave Optoelectronics Technology Co., Ltd., Longgang, China). The illuminant of D_65_ with 10° observing angle and 8 mm apertures was used. The average lightness, redness, and yellowness at three different points on the fresh incision of the LT muscle was recorded. The average pH of meat at three different points on the fresh incision of the LT muscle was taken at 45 min, 24 h, 48 h, and 72 h after slaughter at 4°C using an acidometer (testo-205; Testo, Lenzkrich, Germany). Water content was determined by vacuum freeze drying [10]. Pressing loss was the water loss of meat tissue under 35 kg pressure for 5 min (RH-1000; Guangzhou Runhu Instruments Co., Ltd., Guangzhou, China); cooking loss was the water loss in a water bath at 80°C for 45 min; and drip loss was the water loss in an air-inflatable food bag for 24 h at 0°C to 4°C.

### Fatty acid analysis

Total lipids were extracted from LT tissue samples following Folch et al [14]. Briefly, 0.1 g of freeze-dried LT tissue was placed into 6 mL of chloroform: methanol (2:1, v/v) solvent for 8 h at 4°C and filtered. Then 1 mL of 0.9% NaCl solution was added, layered, and the underlying organic solution was placed into a 1.5 mL sample bottles. One mL of 5% sulfuric acid methanol solution was added and placed in a water bath for 1 h for hydrolysis and methylation. N-hexane extracted the esterified sample 2 to 3 times, n-hexane was blown dry by a nitrogen blower and then, 1 mL of n-hexane was added before storing at −18°C. The fatty acids were analyzed by a gas chromatograph (GC-MS; Agilent 7890A-5975C, Santa Clara, CA, USA) equipped with a HP 88 capillary chromatographic column (length 60 m, internal diameter 250-μm, film thickness 0.2-μm). The temperature regimen of the column was 100°C for 5 min with a subsequent increase to 230°C at 5°C/min. The injector and detector were maintained at 250°C, the carrier gas was nitrogen, and the sample injection volume was 1 μL. The peak value of each fatty acid was identified by using a commercial mixture of 37 standard fatty acids (37 FAME mix, CRM 47885). Fatty acids are expressed as mg per 100 g LT tissue.

The ratios of polyunsaturated fatty acids (PUFA) to saturated fatty acids (SFA) and n-6 to n-3 PUFA, and the atherogenic (AI) and thrombogenic (TI) indices were used to evaluate the health value of the fatty acids. AI and TI were calculated following Ulbricht and Southgate [15]:


$$\begin{array}{l} \text{AI}=(\text{C}12:0+4\times \text{C}14:0+\text{C}16:0)/(\Sigma \text{MUFA}+\Sigma \text{n-}6\text{PUFA}+\Sigma \text{n-}3\text{PUFA});\\ \text{TI}=(\text{C}14:0+\text{C}16:0+\text{C}18:0)/[0.5\times \Sigma \text{MUFA}+0.5\times \Sigma \text{n-}6\text{PUFA}+3\times \Sigma \text{n-}3\text{PUFA}+(\text{n-}3)/(\text{n-}6)] \end{array}$$

where MUFA is monounsaturated fatty acids.

### Amino acid analysis

Amino acids of LT tissue were analyzed using an amino acid analyzer (A300; MembraPure, Bodenheim, Germany). Briefly, 0.1 g LT tissue was placed in a beaker and 10 mL hydrochloric acid (6 mol/L) and 4 drops of phenol solution were added. The mixture was frozen (3 min) and vacuumed (5 min), nitrogen was added, and the mixture was hydrolyzed for 22 h. The hydrolysate was filtered to a constant volume in a 50 mL bottle, and 1 mL filtrate was removed and dried in vacuum, dissolved with 1 mL sodium citrate buffer, and filtered through a 0.22-μm membrane. The ion-exchange chromatographic column (650-0042; MembraPure, Germany) was used, where the amino acids were eluted by a natirum buffer system. After reacting with ninhydrin, the resultant derivatives were measured by UV at wavelengths of 440 and 570 nm simultaneously. Due to a low response at 570 nm, the derivative of Pro was detected at 440 nm. Amino acid quantities were determined by comparison with the retention times and peak areas of a mixture of 16 standard amino acids (C1707015; MembraPure, Germany).

### Statistical analysis

The effects of supplementary grapeseeds (added and not added) and sweet sorghum (silage and hay) and their interactions on carcass parameters and meat quality in lambs were analyzed using the generalized linear models (SPSS 22.0; SPSS Inc., Chicago, IL, USA), with the animal initial weight as the random effect. Difference between means were assessed using the Tukey’s honest significant difference test. Significance was accepted at p<0.05 and as tended to differ at 0.05<p<0.10.

## RESULTS

### Carcass evaluation and meat quality

There was no effect (p>0.05) of the grapeseeds×sweet sorghum interaction on dry matter intake (DMI) and carcass parameters (Table 3). Dry matter intake of the lambs consuming sweet sorghum silage was greater than the lambs consuming sweet sorghum hay (p<0.05). There was a tendency (p = 0.068) of the HCW to be greater in lambs consuming sweet sorghum silage than hay, and a tendency (p = 0.081) of the dressing percentage to be lower in lambs consuming grapeseeds.

The interaction between sweet sorghum×grapeseeds affected (p<0.001) pH_24 h_ and tended to affect (p = 0.068) the lightness of meat (Table 4). The water content and pH_72 h_ of the meat were greater (p<0.05) in lambs consuming sweet sorghum silage than sweet sorghum hay. Meat from lambs with supplementary grapeseeds had greater (p<0.05) redness and tended (p = 0.065) to have greater yellowness and pH_72 h_ than lambs without grapeseeds. The pH of LT muscle declined rapidly (p<0.05) between 45 min and 24 h, and then stabilized (p>0.05).

### Amino acid composition

The interaction between sweet sorghum×grapeseeds affected (p<0.05), essential amino acids (EAA), valine (Val), l-isoleucine (Ile) and lysine (Lys) and tended (p<0.10) to affect aspartic (Asp), l-threonine (Thr) and leucine (Leu) (Table 5). Non-essential amino acids (NEAA), total amino acids (TAA) and the EAA:NEAA ratio of meat were not affected (p>0.05) by dietary treatment. Meat from lambs with supplementary grapeseeds tended (p = 0.075) to have a lower methionine (Met) concentration than lambs without grapeseeds.

### Fatty acid composition

The interaction between sweet sorghum×grapeseeds affected (p<0.05) SFA (C14:0, C15:0, C16:0, C17:0, C18:0, C20:0), UFA (C16:1, C17:1, C18:1c9, C18:2n-6c, C20:3n-6, C20:5n-3) and MUFA, and tended (p = 0.061) to affect PUFA, (n-3 PUFA and n-6 PUFA) (Table 6). Concentrations of C12:0, C22:0, C23:0, C24:0, C18:3n-3, C20:4n-6, C22:6n-3, and the ratio of n-6:n-3 PUFA were greater (p<0.05) and concentrations of C10:0, C20:2 and the ratio of PUFA:SFA tended (p<0.10) to be greater in meat of lambs consuming sweet sorghum hay than lambs consuming sweet sorghum silage. The meat of lambs consuming supplementary grapeseeds tended to have a greater (p = 0.051) concentration of C22:6n-3 and a lower (p = 0.090) TI than meat of lambs not conuming grapeseeds. In addition, meat of the sweet sorghum silage+ grapeseeds (SSG) group had the lowest SFA content, especially C15:0, C20:0, C22:0, C23:0, and C24:0 (p<0.05).

## DISCUSSION

### Carcass evaluation

Grape pomace supplementation can increase lamb productivity without compromising meat quality [7]. A previous study on Small-tailed Han lambs showed that supplementary grapeseeds did not affect DMI, and lambs consuming sweet sorghum silage had higher DMI than lambs consuming sweet sorghum hay [16]. Studies have reported that consumption of tannins had a negative effect on the carcass parameters of lambs [17]. The sweet sorghum with supplementary grapeseeds tended to decrease the dressing percentage but did not affect other carcass parameters. The growth of these lambs was not affected by the intake of grapseeds.

The GR tissue fat depth for all dietary treatments (21.2 to 25.9 mm) was substantially greater than the 5 mm minimum recommended for lambs [18]. The lambs consuming sweet sorghum and grapeseeds had a backfat thickness between 6.35 and 8.34 mm, values which were similar to the 7.2 mm in lambs fed a ratio of 70 to 30 alfalfa to linseed pellet diet [19]. The GB/T 9961-2008 (National Standards of the People’s Republic of China) divided the carcass quality of lambs into four grades, with the best quality having a carcass weight>18 kg, GR tissue depth>14 mm and backfat thickness>5 mm. Therefore, according to these criteria, the carcass of all groups in the present study met the standards of high-quality lamb meat.

### Meat quality

Low pressing loss and drip loss indicates a higher water holding capacity (WHC) and high drip loss results in the loss of water-soluble proteins, vitamins, and other nutrients [20]. No effect of dietary treatment was observed in pressing loss, drip loss and cooking loss, which ranged between 14.7% and 18.8%, 1.70% and 2.22%, and 34.9% and 37.7%, respectively, but these values were considerably lower than in other reports [7,21]. Chickwanha et al [7] reported that the cooking loss of lamb meat showed a negative quadratic response of first decreasing and then increasing with an increase of supplementary grape pomace, and the lowest value was 37.7%.

The WHC, that is, the ability of fresh meat to retain water during cutting, heating, grinding, pressing, and during transport, storage and cooking [22], is an important measurement in evaluating the quality in meat processing, as it indicates the juiciness of the fresh meat and the profitability of the meat processing industry [23]. Meat from SSG lambs had the highest water content, and from lambs fed sweet sorghum silage had a higher water content than meat from lambs fed sweet sorghum hay. These results indicated that meat from lambs fed sweet sorghum silage with grapeseeds had better WHC than the other groups, but the optimal level of grapeseeds requires more research.

Meat color plays an important role as it reflects meat freshness and wholesomeness and is often the only feature consumers consider at the time of purchase [24]. Supplementary grapeseeds increased meat redness and tended to increase yellowness, which enhances the consumers’ positive visual perception of the meat. The change in meat color from red to brown reflects the deterioration of meat and is related to an increase in met-myoglobin concentration as there is an inverse correlation between met-myoglobin and redness in meat in lambs [25]. The ferric ion in met-myoglobin causes the brown color in the meat, which discourages consumers from buying the meat. Met-myoglobin reductase activity in mitochondria reduces met-myoglobin to oxy-myoglobin by reducing Fe^3+^ to Fe^2+^, and in this way maintains the red color longer [26]. Free radicals disrupt the function of mitochondria, but elevated levels of antioxidants protect the mitochondria, resulting in an increased redness in the meat. Grapeseeds are rich in tannins, anthocyanins and other bioactive substances that possess high antioxidant capacity [27], which may explain the greater redness in the meat in lambs consuming grapeseeds. Further support was provided by Luciano et al [28], who reported that supplementary tannins fed to lambs increased meat redness. In addition, meat redness correlated negatively with the sensory degradation of odor and color, while yellowness correlated positively with sensory degeneration [29].

It was reported that the pH of meat after slaughter was related to pre-slaughter stress, and that an increase in stress intensity slows down the pH decline rate [30]. Glycolysis is one of the major biochemical processes that regulates pH [31]. Glycogen, the main energy source of muscles, is converted to ATP, lactic acid, and finally to hydrogen ions [32]. Pre-slaughter stress causes tension, anxiety and excitement in livestock and increases energy depletion, which eventually leads to a decrease in muscle glycogen content post-mortem, and a decrease in pH [33]. In this study, the post-mortem pH of LT muscle decreased from 6.59 to 6.67 at 45 min post-mortem to 5.44 to 5.71 at 24 h post-mortem, which was close to the acceptable final meat pH value of 5.4 to 5.7 [31]. The pH values in this study indicated that the slaughter process effectively minimized stress of the lambs and the decrease of muscle glycogen reserves.

### Amino acid composition

Amino acids provide nutrients and flavor, which are important parameters for meat quality. The addition of grapeseeds tended to decrease methionine (Met) content in the meat, which may be related to the tannins in grapeseeds. Tannins reduce digestibility of feed and of Met and enhances the synthesis of cystine [34]. Glutamate (Glu), aspartic acid (Asp), lysine (Lys), and leucine (Leu) were the most abundant amino acids for all diets in this study, which is consistent with other reports [21]. Glu and Asp are important amino acids for flavor [35], while Lys and Leu are essential amino acids for humans. The ratio of EAA to TAA in meat for all diets ranged between 0.392 and 0.399, which was close to the 0.40 recommended by FAO [36]. The EAA:NEAA ratio for all lambs was greater than the minimum of 0.60 recommended by FAO’s [36] for healthy meat. This indicates that lamb meat produced by feeding sweet sorghum with supplementary grapeseeds meets the demand for high quality protein.

### Fatty acid composition

Fatty acid composition is emerging as a key factor in determining meat quality, prompting nutritionists to strive towards improving beneficial meat fatty acid concentrations in ruminants [37]. Saturated fat intake increases low density lipoprotein cholesterol in the blood, which is associated with an increased risk of cardiovascular diseases [38]. The most abundant fatty acids in the lamb meat in the present study were oleic acid, palmitic acid, and stearic acid, consistent with the fatty acid composition of lambs [37]. Lambs consuming sweet sorghum silage had lower SFA in meat than lambs consuming sweet sorghum hay, and the sweet sorghum silage plus grapeseeds group had the lowest SFA content. The interaction between grapeseeds and sweet sorghum affected most SFA. The mechanism is not clear, but the interaction clearly had a more favorable effect on fatty acid composition in the silage than the hay. The PUFA:SFA ratio of meat in this study ranged between 0.12 and 0.21, which was similar to values reported for lambs by Enser et al [39], but below the minimum ratio of 0.4 recommended for healthy meat [40].

The AI and TI are used to assess meat quality, as they indicate the impact of fatty acid composition on human health, in particular the likelihood of atherosclerosis and coronary thrombosis [14]. An AI of less than 1 is considered acceptable for human health [41]. In this study, all but the lambs consuming sweet sorghum hay without supplementary grapeseeds (1.07) were within the recommended healthy range. Grapeseeds tended to decrease TI, indicating that meat from lambs fed supplementary grapeseeds was less likely to lead to coronary thrombosis.

The n-3 PUFA, particularly eicosapentaenoic acid (EPA) and docosahexaenoic acid (DHA), are associated with cardiovascular health benefits [42]. However, a high ratio of n-6:n-3 PUFA can contribute to thrombosis and pro-inflammation, which can lead to atherosclerosis, obesity and diabetes [43]. In the present study, the n-6:n-3 PUFA ratios of meat ranged between 6.94 and 8.93, which were higher than the ratio of up to 4.0 recommended by the British Department of Health [44], but within the range between 5 and 10 recommended by FAO [45] for healthy meat. The n-6:n-3 PUFA ratio of meat from lambs consuming sweet sorghum silage was lower than the meat from lambs consuming sweet sorghum hay, which was attributed mainly to a greater decrease in n-6 PUFA than in n-3 PUFA. Although sweet sorghum silage reduced n-3 PUFA, especially EPA and DHA, the proportion of fatty acid composition was altered to be healthier. Of importance was that supplementary grapeseeds increased cis-8, 11, 14-eicosatrienoic acid (C20:3n-6) and tended to increase EPA and DHA, which indicated that grapeseeds can improve the composition of beneficial fatty acids.

## CONCLUSION

Supplementary grapeseeds increased muscle redness, which was most likely related to the tannins and antioxidant activity of the grapeseeds. Consumers associate meat color with freshness, and consequently, this enhanced the positive perception of the meat to consumers. Meat from lambs consuming sweet sorghum silage contained a lower ratio of n-6:n-3 PUFA than meat from lambs consuming sweet sorghum hay, which was attributed to a decrease of linoleic acid (C18:2n-6c), cis-8, 11, 14-eicosatrienoic acid (C20:3n-6) and arachidonic acid (C20:4n-6). Meat from lambs consuming sweet sorghum silage had a healthier fatty acid composition than from lambs consuming sweet sorghum hay, and meat from lambs consuming supplementary grapeseeds tended to have a lower thrombogenic index than meat from lambs not consuming grapeseeds. Supplementary grapeseeds, especially with sweet sorghum silage improved the composition of beneficial fatty acids, notably EPA and DHA. In conclusion, the present study provides evidence that feeding lambs sweet sorghum with supplementary grapeseeds improves meat color and fatty acids compositions and produces high quality meat. Further research is needed to determine the optimal amount of grapeseeds to be supplemented.

## Figures and Tables

**Table 1:** Composition of sweet sorghum silage and hay, alfalfa silage and grapeseeds

Item (g/kg, DM basis)	Sweet sorghum silage	Sweet sorghum hay	Alfalfa silage	Grapeseeds
CP	55.8	50.6	137.2	89.6
EE	17.8	17.3	24.4	22.1
Ash	58.6	54.0	144.5	31.2
ADF	290	390	404	477
NDF	535	682	520	556
NFC^1)^	333	196	174	381
Tannins	-	-	-	24.2
ME^2)^ (MJ/kg)	18.8	18.7	19.1	28.6

CP, crude protein; EE, ether extract; ADF, acid detergent fiber; NDF, neutral detergent fiber; NFC, non-fiber carbohydrate; ME, metabolizable energy; DM, dry matter.

1) Non-fiber carbohydrate (NFC) = 100− (NDF+CP+EE+ash)%.

2) Calculated according to Tables of Feed Composition and Nutritive Values in China (Chinese Feed Database, 2009).

**Table 2:** Ingredients and chemical composition of the concentrate feed

Item (g/kg, DM basis)	Experimental diets	

	Concentrate without grapeseeds	Concentrate with grapeseeds
Ingredients		
Corn	600	560
Wheat bran	80.0	60.0
Soybean meal	150	150
Cottonseed meal	80.0	80.0
Grapeseed	-	60.0
Stone power	10.0	10.0
NaCl	20.0	20.0
Commercial premix^1)^	60.0	60.0
Nutrient levels		
ME (MJ/kg)	1.19	1.20
OM	91.4	91.3
CP	141	149
EE	13.6	14.8
NDF	269	326
ADF	57.6	77.0

DM, dry matter; ME, metabolizable energy; OM, organic matter; CP, crude protein; EE, ether extract; NDF, neutral detergent fiber; ADF, acid detergent fiber.

1) Premix provided the following per kilogram diet: 1,700 IU vitamin A; 190 IU vitamin D; 18 IU vitamin E; 17 mg Cu; 40 mg Zn; 70 mg Fe; 38 mg Mn; 1.5 mg I; 0.30 mg Co; 0.28 mg Se.

**Table 3:** Effect of grapeseeds (G) and sweet sorghum forage (F) on dry matter intake and carcass parameters in Small-tailed Han lambs

Item	Dietary treatment^1)^				SEM	p-value^2)^		
	
	SS	SSG	SH	SHG		F	G	F×G
Dry matter intake (g/d)	1,249^ab^	1,259^a^	1,050^b^	1,176^ab^	28.46	0.003	0.121	0.394
Concentrate dry matter intake (g/d)	550	564	521	530	17.5	0.305	0.912	0.861
Live body weight (kg)	39.0	41.3	35.8	38.2	1.128	0.123	0.262	0.970
Hot carcass weight (kg)	19.3	20.1	17.6	17.8	0.602	0.068	0.647	0.809
Dressing percentage (%)	49.5	48.8	49.1	46.6	0.517	0.153	0.081	0.341
Rib eye area (cm^2^)	17.2	18.6	16.4	16.4	0.785	0.329	0.629	0.631
GR tissue depth (mm)	25.9	23.4	21.2	22.2	1.042	0.130	0.707	0.382
Backfat thickness (mm)	8.34	7.08	6.76	6.35	0.439	0.159	0.310	0.607

SEM, standard error of the mean.

1) SS, sweet sorghum silage; SSG, sweet sorghum silage+grapeseeds; SH, sweet sorghum hay; SHG, sweet sorghum hay+grapeseeds;

2) F, sweet sorghum forage (silage vs hay); G, grapeseeds (with vs without); F×G, the interaction between sweet sorghum forage and grapeseeds.

a,b Means within a row with different lowercase letters differ from each other (p<0.05).

**Table 4:** Effect of grapeseeds (G) and sweet sorghum forage (F) on meat quality in Small-tailed Han lambs

Item	Dietary treatment^1)^				SEM	p-value^2)^		
	
	SS	SSG	SH	SHG		F	G	F×G
Water content (%)	70.8^ab^	71.4^a^	68.9^bc^	67.9^c^	0.401	<0.001	0.722	0.158
Pressing loss (%)	18.8	17.3	17.7	14.7	0.773	0.182	0.113	0.597
Drip loss (%)	1.70	2.17	1.92	2.22	0.128	0.585	0.106	0.732
Cooking loss (%)	34.9	37.1	37.7	36.5	0.710	0.431	0.685	0.200
Lightness (L*)	29.2	30.6	30.3	29.3	0.354	0.897	0.735	0.068
Redness (a*)	6.13	6.69	6.24	7.41	0.200	0.223	0.012	0.375
Yellowness (b*)	9.06	10.0	9.96	10.2	0.186	0.110	0.065	0.245
pH_45 min_	6.59^A^	6.60^A^	6.60^A^	6.67^A^	0.050	0.635	0.656	0.787
pH_24 h_	5.45^Bb^	5.87^Ba^	5.73^Bab^	5.48^Bab^	0.059	0.574	0.358	<0.001
pH_48 h_	5.61^B^	5.55^B^	5.55^B^	5.83^B^	0.066	0.375	0.363	0.169
pH_72 h_	5.56^Bab^	5.71^Ba^	5.44^Bb^	5.49^Bab^	0.037	0.004	0.096	0.414
SEM of pH at different times	0.110	0.091	0.098	0.119	-	-	-	-
p-value of pH at different times	<0.001	<0.001	<0.001	<0.001	-	-	-	-

SEM, standard error of the mean.

1) SS, sweet sorghum silage; SSG, sweet sorghum silage+grapeseeds; SH, sweet sorghum hay; SHG, sweet sorghum hay+grapeseeds.

2) F, sweet sorghum forage (silage vs hay); G, grapeseeds (with vs without); F×G, the interaction between sweet sorghum forage and grapeseeds.

A,B Means of pH within a column with different capital letters differ from each other (p<0.05).

a–c Means within a row with different lowercase letters differ from each other (p<0.05).

**Table 5:** Effect of grapeseeds (G) and sweet sorghum forage (F) on amino acid profiles of Small-tailed Han lamb meat (g/100 g *Longissimus thoracis* tissue)

Item	Dietary treatment^1)^				SEM	p-value^2)^		
	
	SS	SSG	SH	SHG		F	G	F×G
NEAA	10.9	11.3	11.2	10.5	0.233	0.519	0.613	0.218
Asp	1.93	2.02	2.01	1.85	0.040	0.520	0.590	0.083
Ser	0.70	0.72	0.71	0.69	0.017	0.777	0.985	0.559
Glu	3.11	3.21	3.19	2.97	0.066	0.507	0.654	0.193
Gly	0.83	0.85	0.87	0.79	0.019	0.807	0.414	0.133
Ala	1.09	1.11	1.12	1.02	0.023	0.452	0.329	0.114
Tyr	0.68	0.66	0.66	0.65	0.015	0.690	0.600	0.980
His	0.90	0.89	0.88	0.80	0.026	0.235	0.330	0.427
Arg	1.18	1.24	1.20	1.14	0.029	0.474	0.920	0.263
Pro	0.53	0.56	0.55	0.55	0.014	0.928	0.716	0.523
EAA	7.20	7.44	7.42	6.73	0.135	0.296	0.339	0.045
Thr	0.85	0.89	0.89	0.82	0.017	0.712	0.596	0.078
Val	0.98	1.03	1.00	0.92	0.018	0.129	0.554	0.032
Met	0.31	0.28	0.34	0.27	0.015	0.847	0.075	0.542
Ile	0.88	0.91	0.89	0.80	0.017	0.096	0.336	0.028
Leu	1.54	1.59	1.58	1.44	0.031	0.319	0.467	0.073
Phe	0.94	0.95	0.96	0.88	0.016	0.365	0.252	0.120
Lys	1.70	1.79	1.76	1.60	0.035	0.301	0.511	0.039
TAA	18.2	18.7	18.6	17.2	0.365	0.430	0.505	0.131
EAA:TAA ratio	0.397	0.398	0.399	0.392	0.002	0.426	0.344	0.121
EAA:NEAA ratio	0.66	0.66	0.66	0.64	0.004	0.434	0.349	0.117

SEM, standard error of the mean; NEAA, non-essential amino acids; EAA, essential amino acids; TAA, total amino acids.

1) SS, sweet sorghum silage; SSG, sweet sorghum silage+grapeseeds; SH, sweet sorghum hay; SHG, sweet sorghum hay+grapeseeds.

2) F, sweet sorghum forage (silage vs hay); G, grapeseeds (with vs without); F×G, the interaction between sweet sorghum forage and grapeseeds.

**Table 6:** Effect of grapeseeds (G) and sweet sorghum forage (F) on fatty acid profiles of Small-tailed Han lamb meat (mg/100 g *Longissimus thoracis* tissue)

Item	Dietary treatment^1)^				SEM	p-value^2)^		
	
	SS	SSG	SH	SHG		F	G	F×G
SFA	581	343	440	664	58.2	0.371	0.943	0.022
C10:0	3.15	3.04	3.18	3.83	0.141	0.095	0.267	0.115
C12:0	2.81	2.69	2.91	3.19	0.084	0.040	0.580	0.175
C14:0	24.3	14.6	17.9	28.6	2.49	0.381	0.900	0.017
C15:0	3.20^ab^	2.61^b^	3.44^ab^	4.58^a^	0.259	0.006	0.496	0.033
C16:0	289	176	215	339	29.4	0.384	0.913	0.020
C17:0	12.7	9.03	12.9	18.6	1.35	0.025	0.641	0.031
C18:0	235	124	173	254	24.8	0.442	0.734	0.028
C20:0	3.03^ab^	2.69^b^	3.03^ab^	3.71^a^	0.132	0.013	0.413	0.011
C22:0	2.82^ab^	2.45^b^	3.07^ab^	3.16^a^	0.101	0.002	0.353	0.125
C23:0	1.90^ab^	1.66^b^	2.25^ab^	2.45^a^	0.073	<0.001	0.607	0.054
C24:0	3.29^ab^	2.94^b^	3.69^ab^	3.87^a^	0.100	<0.001	0.367	0.065
UFA	507	307	304	634	56.9	0.510	0.484	0.005
MUFA	447	253	223	523	52.8	0.790	0.550	0.005
C14:1	2.09	3.08	1.86	2.47	0.359	0.535	0.234	0.781
C16:1	12.6	8.84	8.33	14.7	1.24	1.27	0.542	0.022
C17:1	4.90^ab^	3.88^b^	4.48^ab^	7.19^a^	0.463	0.047	0.246	0.010
C18:1c9	427	237	209	498	51.4	0.802	0.563	0.005
PUFA	60.6^b^	54.6^b^	80.1^ab^	111^a^	7.05	<0.001	0.201	0.061
C18:2n-6c	32.4^b^	29.0^b^	41.4^ab^	62.0^a^	4.08	<0.001	0.139	0.040
C18:3n-3	3.44	3.16	3.80	5.79	0.384	0.015	0.164	0.064
C20:2	2.60	1.75	2.87	2.86	0.213	0.056	0.240	0.246
C20:3n-6	2.24^b^	2.18^b^	2.61^ab^	3.76^a^	0.197	<0.001	0.049	0.027
C20:4n-6	16.4^ab^	15.0^b^	25.5^ab^	31.9^a^	2.38	<0.001	0.491	0.281
C20:5n-3	1.80	1.73	1.87	2.83	0.104	0.024	0.090	0.047
C22:6n-3	1.71^b^	1.78^b^	2.02^ab^	2.54^a^	0.091	<0.001	0.051	0.140
n-3 PUFA	6.95	6.67	7.70	11.2	0.631	0.007	0.104	0.055
n-6 PUFA	51.0^b^	46.2^b^	69.6^ab^	97.6^a^	6.49	<0.001	0.213	0.078
n-6:n-3 PUFA ratio	7.63	6.94	8.75	8.93	0.389	0.021	0.707	0.519
PUFA:SFA ratio	0.12	0.17	0.20	0.21	0.017	0.070	0.312	0.472
AI	0.80	0.77	1.09	0.72	0.074	0.354	0.123	0.186
TI	2.05	1.84	2.62	1.79	0.174	0.391	0.090	0.315

SEM, standard error of the mean; SFA, saturated fatty acids; UFA, unsaturated fatty acids; MUFA, monounsaturated fatty acids; PUFA, polyunsaturated fatty acids; AI, atherogenic index; TI, thrombogenic index.

1) SS, sweet sorghum silage; SSG, sweet sorghum silage+grapeseeds; SH, sweet sorghum hay; SHG, sweet sorghum hay+grapeseeds;

2) F, sweet sorghum forage (silage vs hay); G, grapeseeds (with vs without); F×G, the interaction between sweet sorghum forage and grapeseeds.

a,b Means with different lowercase letters within a row differ from each other (p<0.05).
